# Supplementary material for: Hofbauer cell function in the term placenta associates with adult cardiovascular and depressive outcomes
Source: Nat Commun. 2023 Nov 14;14:7120. doi: 10.1038/s41467-023-42300-8 (PMC10645763; doi:10.1038/s41467-023-42300-8)
Supplement: Supplementary file 1 — Supplementary Information [file 41467_2023_42300_MOESM1_ESM.pdf]

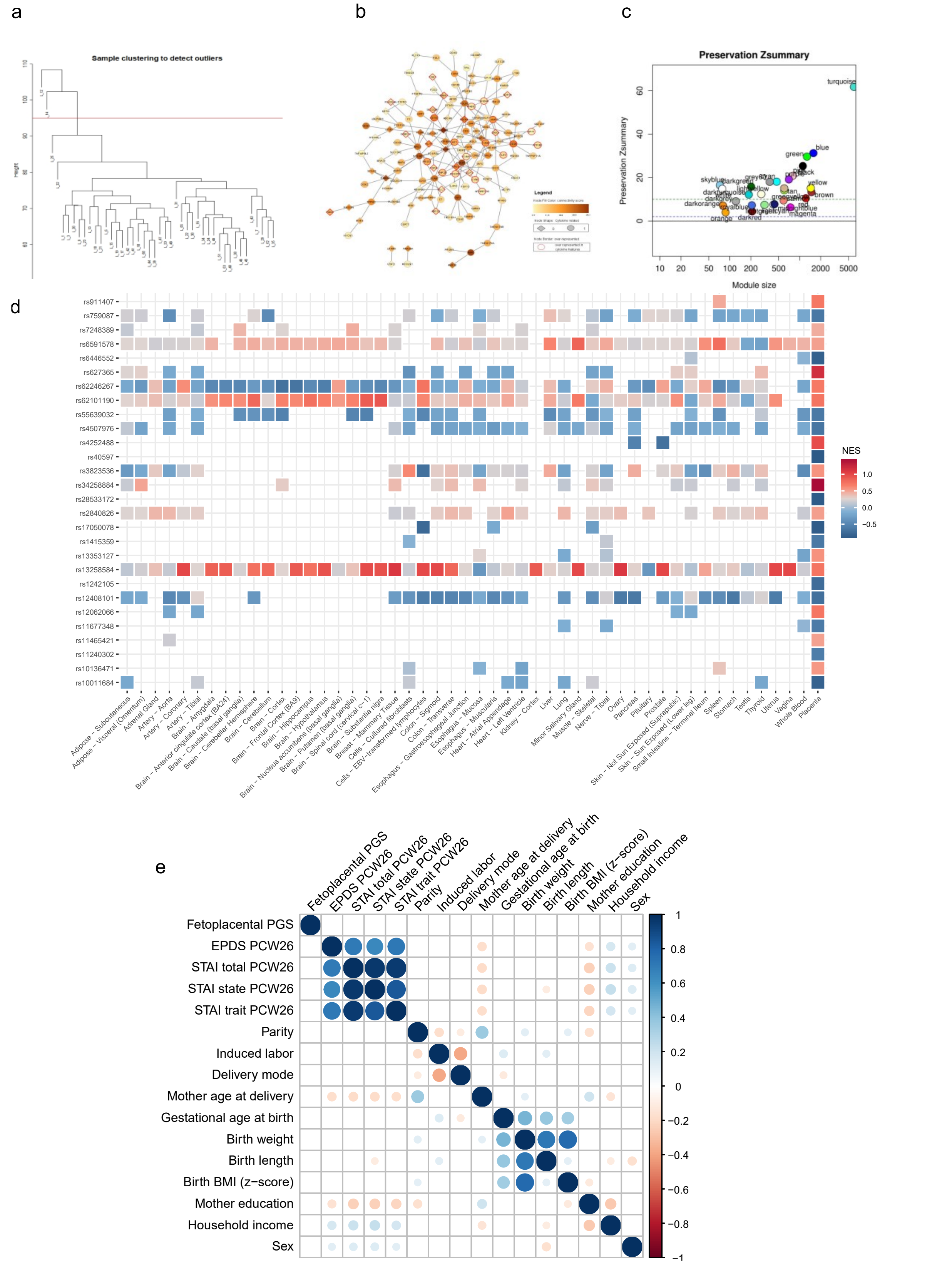

Supplementary Figure 1

WGCNA and fetoplacental PGS . a) Outlier removal with hierarchical clustering of placental villous samples prior to WGCNA. b) Cyan module network with nodes representing genes and color representing connectivity score. c) Preservation analysis of WGCNA modules in Yang *et al.* Above the dashed green line indicates strong evidence of preservation. d) Comparison of eQTLs used to generate the fetoplacental PGS (y-axis) in all GTEx v8 tissues (along x-axis; last value is the placenta eQTLs used in this study for comparison purposes). If an eQTL was identified in that tissue, it is colored with respect to its normalized effect size (NES) as indicated in the GTEx catalogue. e) Correlation plot of the fetoplacental PGS and various perinatal factors. Empty box indicates no significant correlation at an uncorrected p-value threshold of 0.05. Correlations with a p-value <0.05 have a color and size proportional to their r.

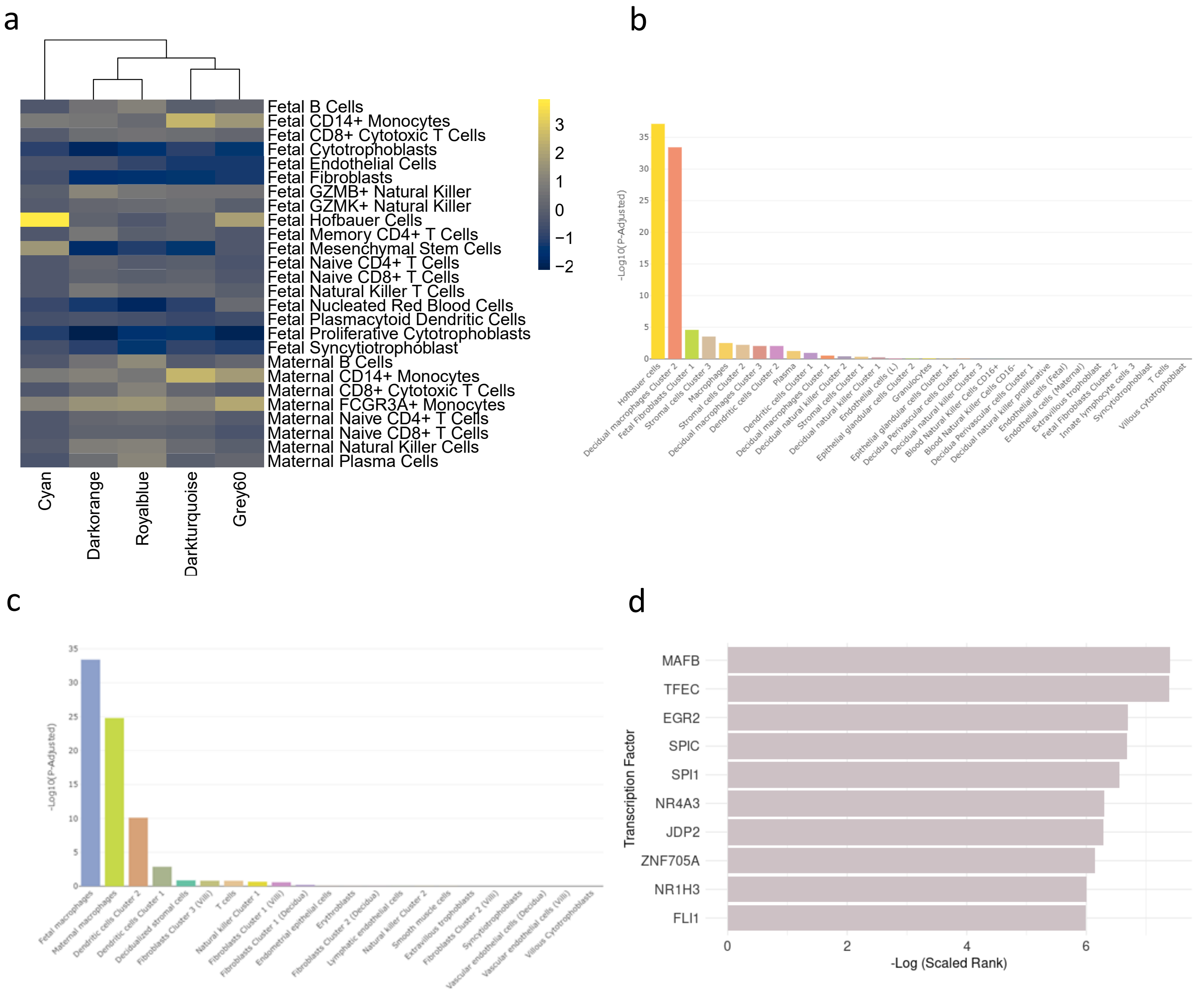

Supplementary Figure 2

Cell type enrichment and transcription factor enrichment of the cyan module. a) Module scores for the inflammation-related modules in scRNA-seq data from Campbell *et al* 2023 b) Enrichment of the cyan module in scRNA-seq data from Vento-Tormo *et al* (b) and Suryawanshi *et al* (c) both show strong enrichment in Hofbauer cells. Note these studies also included maternal macrophages, which are unlikely to be present in our dataset. d) Transcripton factor enrichment analysis of the cyan module using ChEA3 and the Top Rank analysis.

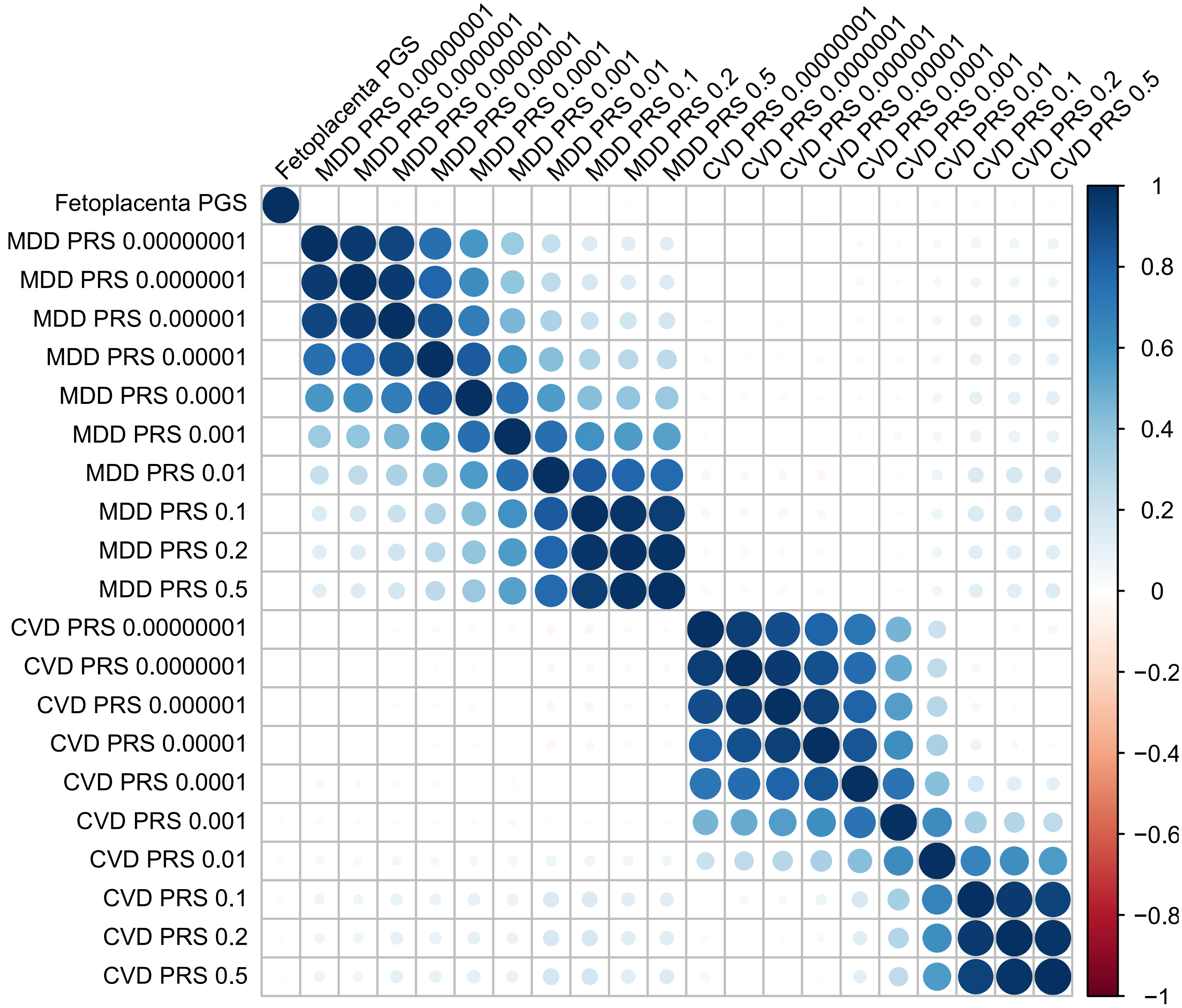

Supplementary Figure 3

Correlation of fetoplacental PGS with major depression disorder (MDD) PRS and cardiovascular disorder (CVD) PRS. Empty box indicates no correlation at an uncorrected p-value threshold of 0.05. Correlations with a p-value<0.05 have a color and size proportional to their r.

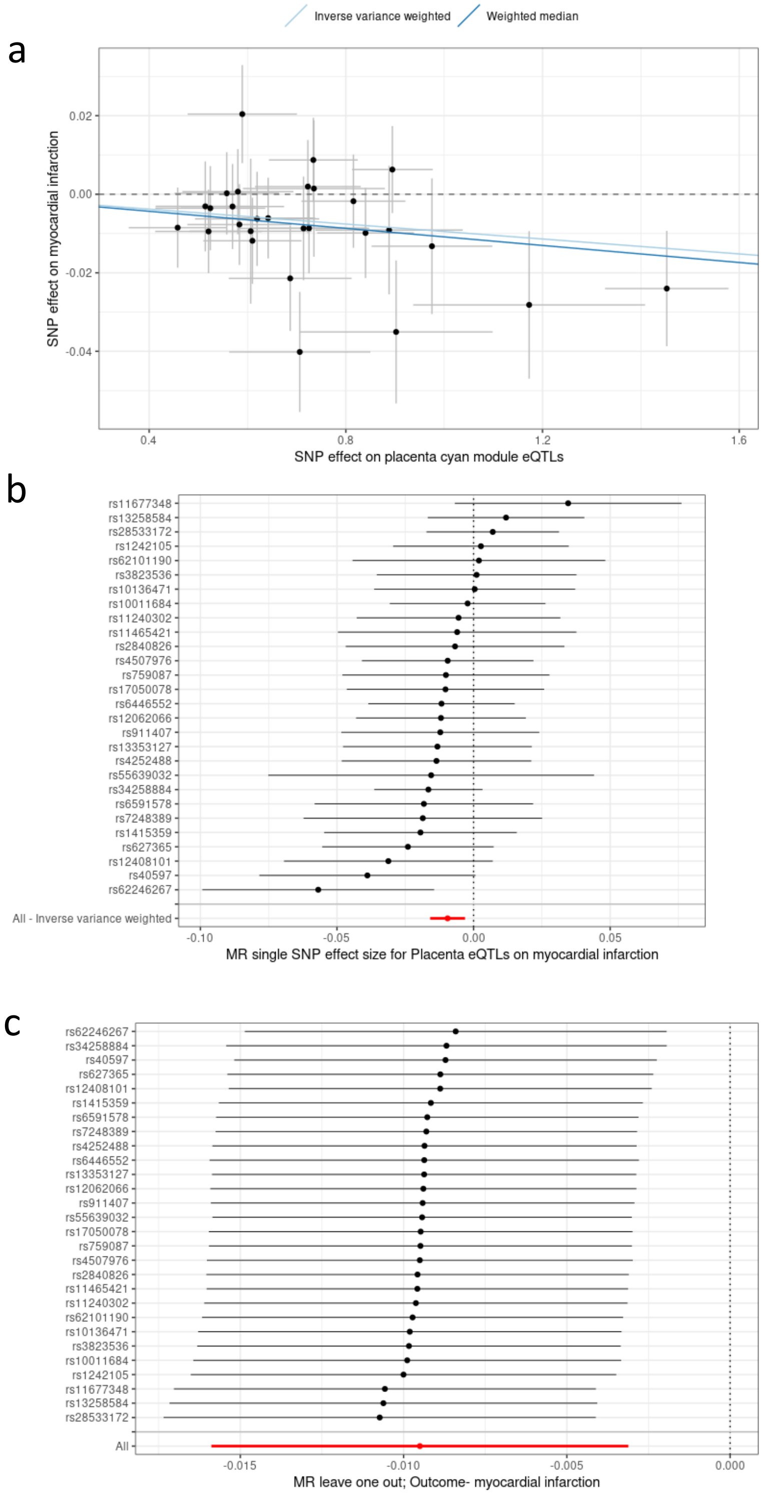

Supplementary Figure 4
Scatterplot, single SNP and leave one out analysis for myocardial infarction (n= 184,305). a) Scatterplot of SNPs with their effect size for cyan module gene expression (x-axis) and myocardial infarction (y-axis). Dark blue line uses the weighted median method, and the light blue line uses the IVW method. b) Single SNP analysis for individual SNPs (in black using the Wald ratio) and combined analysis using the IVW method (red). Data are presented as the IVW estimate +/- 95% confidence intervals. c) IVW results of the analysis when each SNP is sequentially removed from the analysis. Removed SNP indicated on the y-axis, combined IVW for all SNPs is in red. Data are presented as the IVW estimate +/- 95% confidence intervals. IVW; Inverse Variance Weighted.

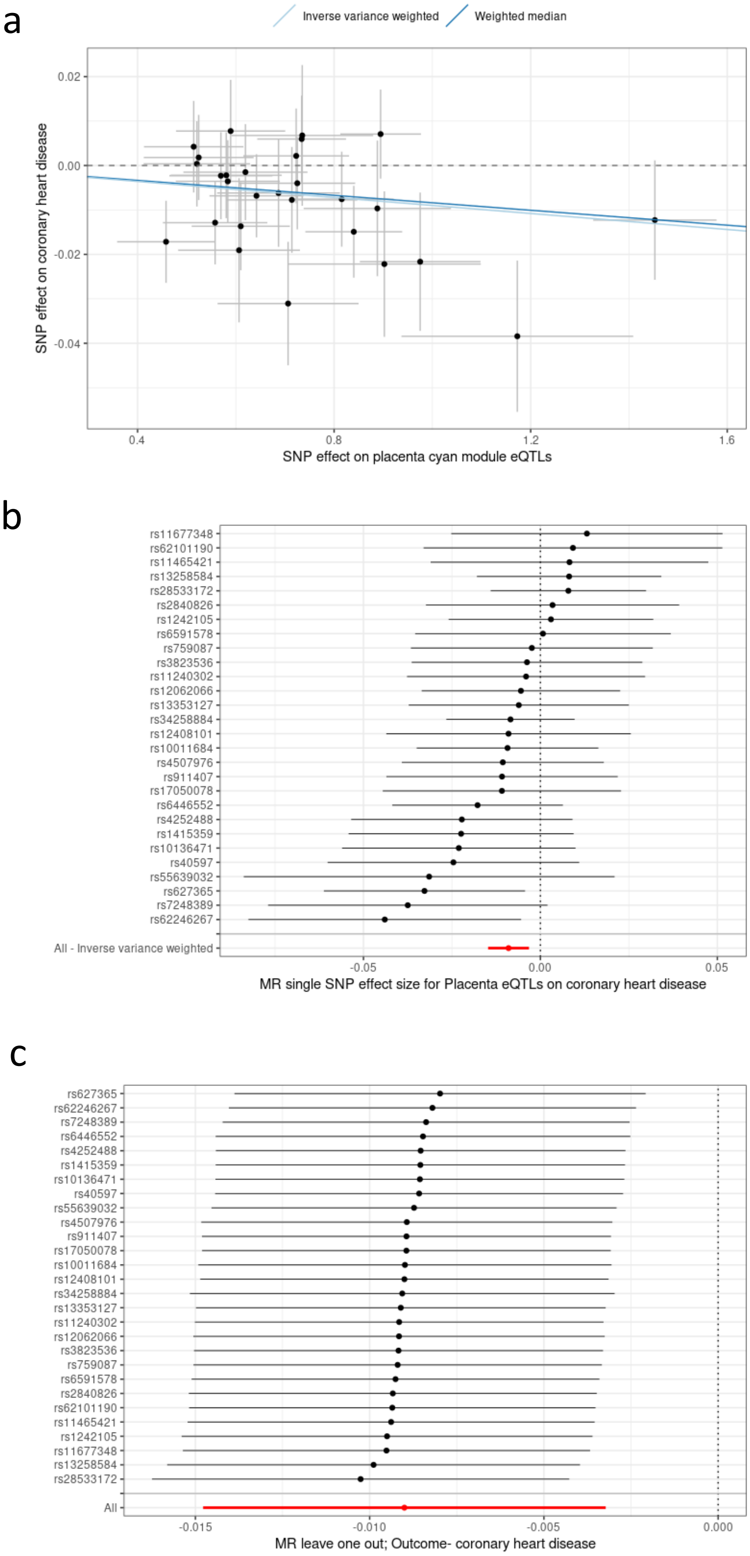

Supplementary Figure 5
Scatterplot, single SNP and leave one out analysis for coronary heart disease (n = 171,875). a) Scatterplot of SNPs with their effect size for cyan module gene expression (x-axis) and coronary heart disease (y-axis). Dark blue line uses the weighted median method, and the light blue line uses the IVW method. b) Single SNP analysis for individual SNPs (in black using the Wald ratio) and combined analysis using the IVW method (red). Data are presented as the IVW estimate +/- 95% confidence intervals. c) IVW results of the analysis when each SNP is sequentially removed from the analysis. Removed SNP indicated on the y-axis, combined IVW for all SNPs is in red. Data are presented as the IVW estimate +/- 95% confidence intervals. IVW; Inverse Variance Weighted.

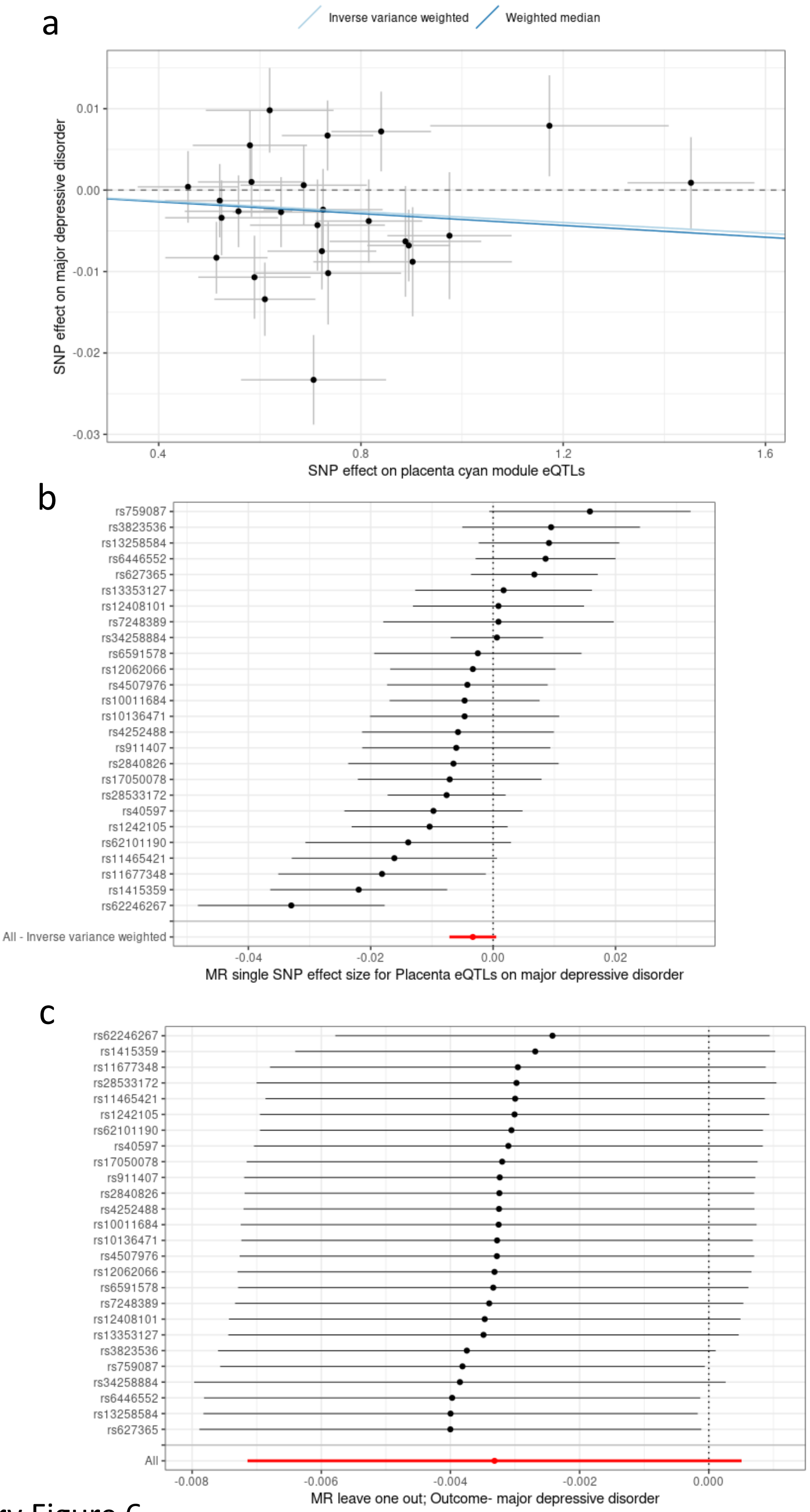

Supplementary Figure 6

Scatterplot, single SNP and leave one out analysis for major depressive disorder (n = 500,199). a) Scatterplot of SNPs with their effect size for cyan module gene expression (x-axis) and major depressive disorder (y-axis). Dark blue line uses the weighted median method, and the light blue line uses the IVW method. b) Single SNP analysis for individual SNPs (in black using the Wald ratio) and combined analysis using the IVW method (red). Data are presented as the IVW estimate +/- 95% confidence intervals. c) IVW results of the analysis when each SNP is sequentially removed from the analysis. Removed SNP indicated on the y-axis, combined IVW for all SNPs is in red. Data are presented as the IVW estimate +/- 95% confidence intervals. IVW; Inverse Variance Weighted.

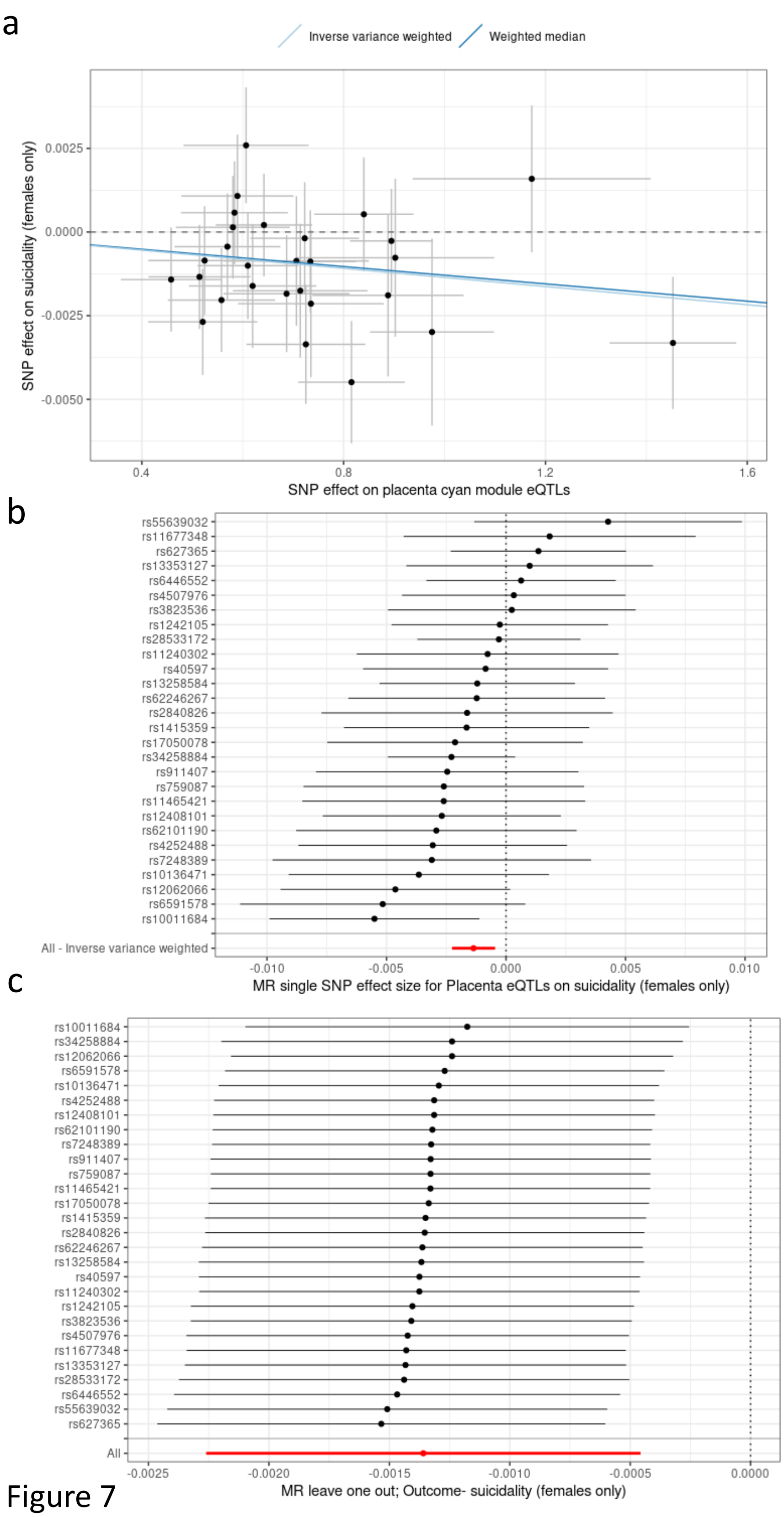

Supplementary Figure 7

Scatterplot, single SNP and leave one out analysis for suicidality (females only; n = 65,572). a) Scatterplot of SNPs with their effect size for cyan module gene expression (x-axis) and suicidality (females only; y-axis). Dark blue line uses the weighted median method, and the light blue line uses the IVW method. b) Single SNP analysis for individual SNPs (in black using the Wald ratio) and combined analysis using the IVW method (red). Data are presented as the IVW estimate +/- 95% confidence intervals. c) IVW results of the analysis when each SNP is sequentially removed from the analysis. Removed SNP indicated on the y-axis, combined IVW for all SNPs is in red. Data are presented as the IVW estimate +/- 95% confidence intervals. IVW; Inverse Variance Weighted.

a

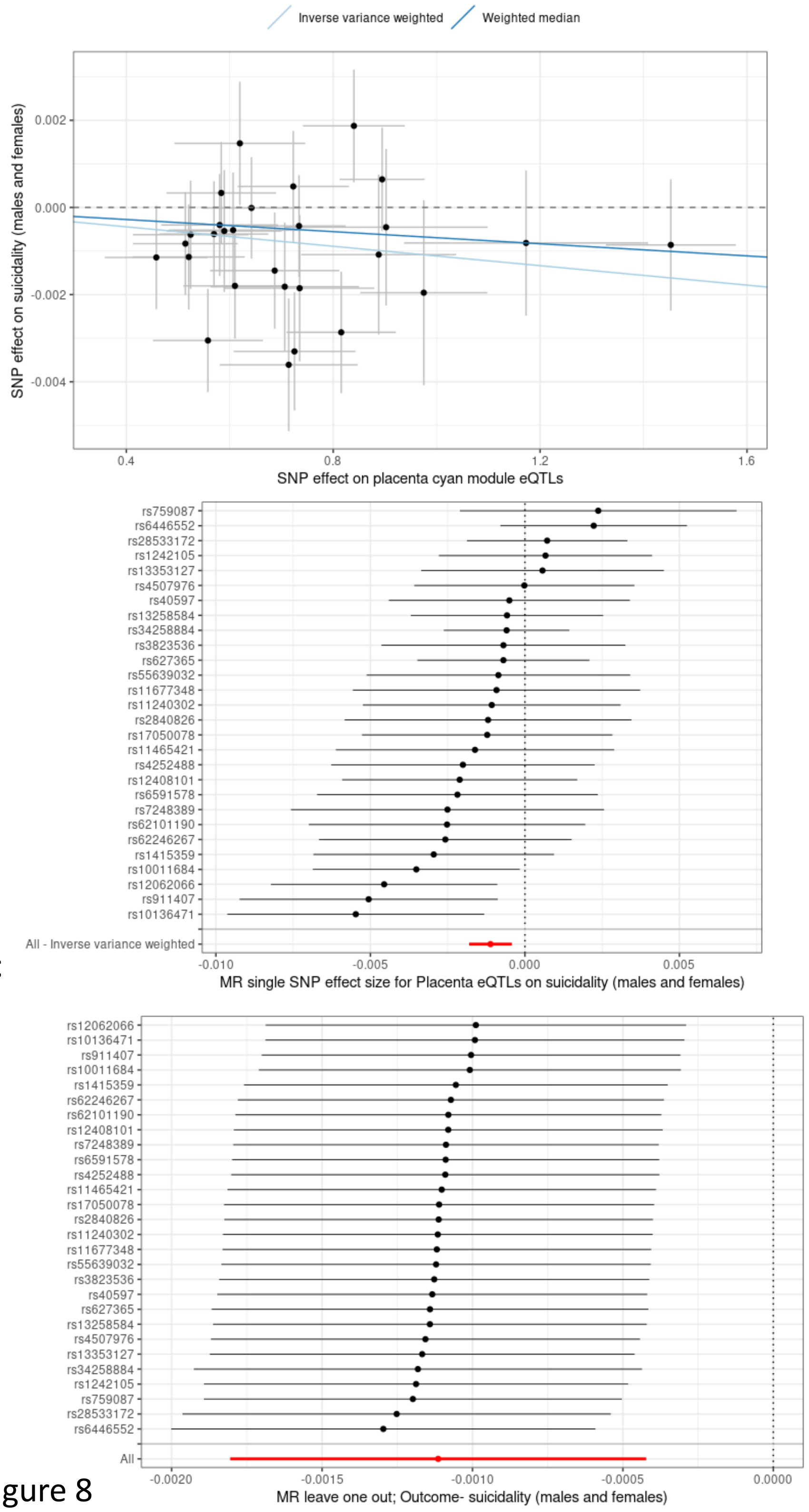

Supplementary Figure 8

Scatterplot, single SNP and leave one out analysis for suicidality (males and females combined; n = 117,177). a) Scatterplot of SNPs with their effect size for cyan module gene expression (x-axis) and suicidality (males and females combines; y-axis). Dark blue line uses the weighted median method, and the light blue line uses the IVW method. b) Single SNP analysis for individual SNPs (in black using the Wald ratio) and combined analysis using the IVW method (red). Data are presented as the IVW estimate +/- 95% confidence intervals. c) IVW results of the analysis when each SNP is sequentially removed from the analysis. Removed SNP indicated on the y-axis, combined IVW for all SNPs is in red. Data are presented as the IVW estimate +/- 95% confidence intervals. IVW; Inverse Variance Weighted.
