# Supplementary material for: Hofbauer cell function in the term placenta associates with adult cardiovascular and depressive outcomes
Source: Nat Commun. 2023 Nov 14;14:7120. doi: 10.1038/s41467-023-42300-8 (PMC10645763; doi:10.1038/s41467-023-42300-8)
Supplement: Supplementary file 3 — Description of Additional Supplementary Files [file 41467_2023_42300_MOESM3_ESM.pdf]

Description of Additional Supplementary Files

File Name: Supplementary Data 1
Description: Clinical characteristics of placental samples used for RNA sequencing

File Name: Supplementary Data 2
Description: WGCNA modules

File Name: Supplementary Data 3
Description: Gene ontology enrichment for WGCNA modules

File Name: Supplementary Data 4
Description: SNPs used to construct UK Biobank and GUSTO fetoplacental PGS

File Name: Supplementary Data 5
Description: eQTLs associated with genes in the cyan module

File Name: Supplementary Data 6
Description: Main effects of cyan module PGS on ssGSEA scores of other modules

File Name: Supplementary Data 7
Description: Genes used for negative control PGS

File Name: Supplementary Data 8
Description: Main effects of PGS on cord blood molecules

File Name: Supplementary Data 9
Description: Assays used to measure molecular content of cord blood

File Name: Supplementary Data 10
Description: UK Biobank pheWAS results: Full sample

File Name: Supplementary Data 11
Description: UK Biobank pheWAS results: Females

File Name: Supplementary Data 12
Description: UK Biobank pheWAS results: Males

File Name: Supplementary Data 13
Description: Inverse variance weighted results

File Name: Supplementary Data 14
Description: Weighted median results

File Name: Supplementary Data 15
Description: Cochran Q test results

File Name: Supplementary Data 16
Description: MR Egger intercept results

File Name: Supplementary Data 17
Description: PHQ-9 details

File Name: Supplementary Data 18
Description: Studies used for enrichment analysis

File Name: Supplementary Data 19
Description: Hub gene analysis in the cyan module
