# Supplementary material for: Hofbauer cell function in the term placenta associates with adult cardiovascular and depressive outcomes
Source: Nat Commun. 2023 Nov 14;14:7120. doi: 10.1038/s41467-023-42300-8 (PMC10645763; doi:10.1038/s41467-023-42300-8)
Supplement: Supplementary file 5 — Reporting Summary [file 41467_2023_42300_MOESM5_ESM.pdf]

nature portfolio

Corresponding author(s): Dr Eamon Fitzgerald
Dr Michael Meaney

Last updated by author(s): Sep 19, 2023

Reporting Summary

Nature Portfolio wishes to improve the reproducibility of the work that we publish. This form provides structure for consistency and transparency in reporting. For further information on Nature Portfolio policies, see our Editorial Policies and the Editorial Policy Checklist.

Statistics

For all statistical analyses, confirm that the following items are present in the figure legend, table legend, main text, or Methods section.

n/a	Confirmed	
☐	☒	The exact sample size (*n*) for each experimental group/condition, given as a discrete number and unit of measurement
☐	☒	A statement on whether measurements were taken from distinct samples or whether the same sample was measured repeatedly
☐	☒	The statistical test(s) used AND whether they are one- or two-sided<br>*Only common tests should be described solely by name; describe more complex techniques in the Methods section.*
☐	☒	A description of all covariates tested
☐	☒	A description of any assumptions or corrections, such as tests of normality and adjustment for multiple comparisons
☐	☒	A full description of the statistical parameters including central tendency (e.g. means) or other basic estimates (e.g. regression coefficient) AND variation (e.g. standard deviation) or associated estimates of uncertainty (e.g. confidence intervals)
☐	☒	For null hypothesis testing, the test statistic (e.g. *F*, *t*, *r*) with confidence intervals, effect sizes, degrees of freedom and *P* value noted<br>*Give P values as exact values whenever suitable.*
☒	☐	For Bayesian analysis, information on the choice of priors and Markov chain Monte Carlo settings
☒	☐	For hierarchical and complex designs, identification of the appropriate level for tests and full reporting of outcomes
☐	☒	Estimates of effect sizes (e.g. Cohen's *d*, Pearson's *r*), indicating how they were calculated

Our web collection on statistics for biologists contains articles on many of the points above.

Software and code

Policy information about availability of computer code

Data collection

Total RNA from the placental samples was extracted from samples using the phenol-chloroform method, followed by large RNA purification using the NucleoSpin miRNA kit (Machery-Nagel, Düren, Germany) as per manufacturer's instructions. RNA concentrations were determined using a Nanodrop spectrophotometer (Thermo Fisher Scientific, Waltham, MA, USA) and RNA integrity number (RIN) was measured using the Agilent 4200 TapeStation System (Santa Clara, CA, USA). Sequencing libraries were prepared from samples with a RIN > 6 at Novogene AIT Genomics (Singapore). In brief, ribosomal RNA was depleted with the Illumina Ribo-Zero Magnetic Kit for Human/Mouse/Rat (San Diego, CA, USA). Library preparation was done using the NEB Next Ultra Directional RNA Library Prep Kit (New England Biolabs, Ipswich, MA, USA), before sequencing was carried out using the Illumina HiSeq platform with a minimum depth of 50 million paired-end 150bp reads.

Molecular characterization of cord blood from the GUSTO cohort (sample size of between 194-251 depending on specific molecule analyzed) was conducted in duplicate using commercially available assays. Samples were randomized across plates and internal controls were used to estimate cross-plate variation. Assays with a coefficient of variation exceeding 20% across internal standards were excluded. Molecular profiles were analyzed using 1 of 3 methods: single molecule array (SIMOA; IL6, IL10, TNFa, IFN gamma, IL4), DropArray (MCP1, TSH, insulin, VEGFA, LH, IgE, FSH, glucagon, IP10, Leptin, MIP1a, CRP, C-peptide, IL1RA, IGFBP7, prolactin, MIP1b, growth hormone, IGFBP3, IL12p40, GLP1) and enzyme-linked immunosorbent assay (ELISA; adiponectin, free testosterone, testosterone). Supplementary Data 9 describes the individual assays. SIMOA measurements were made using the SIMOA HD-1 Analyzer (Quanterix). DropArray measurements were made using the FlexMAP3D bead-based multiplex system (Luminex). Normalization was carried out across plates using a median centring method. Data with readings outside of the assay limits as indicated by the manufacturer were discarded.

Data analysis

All data were analyzed using previously described packages.
Gene ontology analysis was conducted using gprofiler2 v0.2.1 in R.
Cell type expression was conducted using Seurat v3.2.3 in R.

Genotype data were analysed using SHAPEIT v2.837 , GCTA 1.93.2, biomart v2.50.3 and PRSice v2.2.11.b.
RNA-sequencing were analysed using GSVA v1.42.0, FastQC, MultiQC packages, WGCNA v1.72-1
Mendelian randomization was conducted using TwosampleMR v0.5.7
GWAS enrichment was run using MAGMA v1.10
Drug gene interactions were mined using the Drug-Gene Interaction Database v4.2.0
Enrichment analyses were conducted using GeneOverlap v1.30.0
Regression analyses were used to analyze the association between the fetoplacental PGS and cord blood molecules (GUSTO), ssGSEA (GUSTO) and UK Biobank outcomes using lm() or glm() functions.
R v4.1.1 and Rstudio v1.4.1717 were used for these analyses

For manuscripts utilizing custom algorithms or software that are central to the research but not yet described in published literature, software must be made available to editors and reviewers. We strongly encourage code deposition in a community repository (e.g. GitHub). See the Nature Portfolio guidelines for submitting code & software for further information.

Data

Policy information about availability of data

All manuscripts must include a data availability statement. This statement should provide the following information, where applicable:

- Accession codes, unique identifiers, or web links for publicly available datasets
- A description of any restrictions on data availability
- For clinical datasets or third party data, please ensure that the statement adheres to our policy

Access to data from GUSTO and UK Biobank are dependent on approved application to the respective data access committees. All other data generated in this study are provided in the supplementary material.

RNA-seq data for WGCNA module validation https://www.ncbi.nlm.nih.gov/geo/query/acc.cgi?acc=GSE148241

GWAS for Mendelian randomization
UK Biobank — Neale lab. http://www.nealelab.is/uk-biobank/.
Howard, D. M. et al. Genome-wide meta-analysis of depression identifies 102 independent variants and highlights the importance of the prefrontal brain regions. Nat. Neurosci. 2019 https://gwas.mrcieu.ac.uk/datasets/ieu-b-102/
Nikpay, M. et al. A comprehensive 1,000 Genomes-based genome-wide association meta-analysis of coronary artery disease. Nat. Genet. 47, 1121–1130 (2015). https://gwas.mrcieu.ac.uk/datasets/ieu-a-7/ and https://gwas.mrcieu.ac.uk/datasets/ieu-a-798/

Gene lists for enrichment analyses
Placenta enriched genes Gong et al, 2021
Normotensive mother, high PAPP-A Gong et al, 2021
Normotensive mother, abnormal fetal growth velocity Gong et al, 2021
Normotensive mother, abnormal uterine blood flow Gong et al, 2021
Normotensive mother, abnormal umbilical cord flow Gong et al, 2021
Gestational diabetes Sober et al, 2015
Preecmaplsia no IUGR Sober et al, 2015
Preecmaplsia with IUGR Sober et al, 2015
Depression and anxiety Litzky et al, 2018
Maternal depression Litzky et al, 2018
Acute prenatal stressor Nomura et al, 2021
Sexually dimorphic expressed genes Gonzalez et al, 2018
Preterm birth Pereyra et al, 2019
Intra-amniotic infection Motomura et al, 2021
Sterile inflammation Motomura et al, 2021
Maternal immune activation (poly I:C) Zengeler et al, 2023
Listeria monocytogenes Connor et al, 2022

Single cell RNA-seq datasets
Campbell, K. A. et al. Placental cell type deconvolution reveals that cell proportions drive preeclampsia gene expression differences. Commun. Biol. 2023 61 6, 1–15 (2023). https://www.ncbi.nlm.nih.gov/geo/query/acc.cgi?acc=GSE182381
Vento-Tormo, R. et al. Single-cell reconstruction of the early maternal–fetal interface in humans. Nat. 2018 5637731 563, 347–353 (2018). https://placentacellenrich.gdcb.iastate.edu/
Suryawanshi, H. et al. A single-cell survey of the human first-trimester placenta and decidua. Sci. Adv. 4, (2018). https://placentacellenrich.gdcb.iastate.edu/
Lu-Culligan, A. et al. Maternal respiratory SARS-CoV-2 infection in pregnancy is associated with a robust inflammatory response at the maternal-fetal interface. Med 2, 591-610.e10 (2021) https://www.ncbi.nlm.nih.gov/geo/query/acc.cgi?acc=GSE171381

Datasets used in PRS generation:
Hg19 https://www.ncbi.nlm.nih.gov/datasets/genome/GCF_000001405.13/
1000 genomes https://www.internationalgenome.org/data

Research involving human participants, their data, or biological material

Policy information about studies with human participants or human data. See also policy information about sex, gender (identity/presentation), and sexual orientation and race, ethnicity and racism.

Reporting on sex and gender

Throughout this study we define sex genetically. Sex was accounted for in all analyses, using it either as a covariate or to split

Reporting on sex and gender | the sample. The relevant strategy to account for sex in each analysis is detailed throughout the text.

Reporting on race, ethnicity, or other socially relevant groupings | We provide parent-defined participant identifications of ethnicities for our RNA-seq samples (Chinese- 26 samples; 59%, Malay-7 samples; 16%, Indian- 11 samples; 25%). In our genetic analyses we accounted for population stratification produced by distinct genetic ancestries using genetic principal components in our regression analysis, or using GWAS from similar genetic ancestries for Mendelian randomization analysis.

Population characteristics | Covariates included in GUSTO PRS analysis for ssGSEA scores: the first 3 genetic principal components and sex

Covariates included in UK Biobank pheWAS analysis: the first 10 genetic principal components, age, sex, genotype array and assessment center (categorical variable).

Recruitment | Established cohorts were used throughout this study.

Ethics oversight | Ethical approval for GUSTO was granted by the relevant institutional boards (DSRB reference D/09/021 and CIRB reference 2009/280/D) and written informed consent was received from all participating mothers. Approval for the UK Biobank was obtained by the Northwest Multicentre Research Ethics Committee (REC reference 11/NW/0382), the National Information Governance Board for Health and Social Care and the Community Health Index Advisory Group. Access to data used in the current study was obtained under application #41975.

Note that full information on the approval of the study protocol must also be provided in the manuscript.

Field-specific reporting

Please select the one below that is the best fit for your research. If you are not sure, read the appropriate sections before making your selection.

☒ Life sciences ☐ Behavioural & social sciences ☐ Ecological, evolutionary & environmental sciences

For a reference copy of the document with all sections, see nature.com/documents/nr-reporting-summary-flat.pdf

Life sciences study design

All studies must disclose on these points even when the disclosure is negative.

Sample size | The exclusion criteria for RNA sequencing were: antenatal smoking (confirmed with plasma cotinine), maternal BMI greater than 30kg/m2, antenatal fasting glucose greater than 7 mmol/L or 2 hour oral glucose tolerance test result greater than 11.1 mmol/L, hypertensive disorders of pregnancy, birth prior to 37 weeks of gestation and a gestational age and sex-standardized birthweight percentile less than 10%.
The maximum available sample was used for all analysis in established cohorts.

Data exclusions | Observations that constituted a very small percent of the UK Biobank sample would be prone to unstable results. As such variables were excluded from the pheWAS analysis if they had fewer than 500 observations for a particular category.

Replication | The central conclusion of this study is that the expression of inflammatory mediators in the placenta under homeostatic conditions is protective for adult depressive outcomes. We validated this finding once using regression and Mendelian randomization analysis, in overlapping samples. We validate another finding for cardiovascular disease once using similar methods, but the direction of effect changed from increasing the risk (with regression analyses) to decreasing the risk (with Mendelian randomization). This is likely a function of unmeasured confounding in the regression analysis and underscores the power of Mendelian randomization and the importance of orthogonal validation.

Randomization | No randomization was conducted, as this was a population based analysis. Relevant covariates were adjusted for in regression models:
Covariates included in GUSTO PRS analysis for ssGSEA scores: the first 3 genetic principal components and sex

Covariates included in UK Biobank pheWAS analysis: the first 10 genetic principal components, age, sex, genotype array and assessment center (categorical variable).

Blinding | No blinding was conducted in this analysis, as we took an unbiased approach to identifying genes associated with placental inflammation (by using RNA-seq and WGCNA) and adult outcomes associated with this pattern of gene expression (by using a phenome-wide association study). We then followed up specific findings from this unbiased approach.

Reporting for specific materials, systems and methods

We require information from authors about some types of materials, experimental systems and methods used in many studies. Here, indicate whether each material, system or method listed is relevant to your study. If you are not sure if a list item applies to your research, read the appropriate section before selecting a response.

Materials & experimental systems

n/a	Involved in the study
☒	☐ Antibodies
☒	☐ Eukaryotic cell lines
☒	☐ Palaeontology and archaeology
☒	☐ Animals and other organisms
☒	☐ Clinical data
☒	☐ Dual use research of concern
☒	☐ Plants

Methods

n/a	Involved in the study
☒	☐ ChIP-seq
☒	☐ Flow cytometry
☒	☐ MRI-based neuroimaging
